# Optical Coherence Tomography Analysis of Attenuated Plaques Detected by Intravascular Ultrasound in Patients with Acute Coronary Syndromes

**DOI:** 10.4061/2011/687515

**Published:** 2011-09-15

**Authors:** Takashi Kubo, Yoshiki Matsuo, Yasushi Ino, Takashi Tanimoto, Kohei Ishibashi, Kenichi Komukai, Hironori Kitabata, Atsushi Tanaka, Keizo Kimura, Toshio Imanishi, Takashi Akasaka

**Affiliations:** ^1^Department of Cardiovascular Medicine, Wakayama Medical University, 811-1, Kimiidera, Wakayama 641-8510, Japan; ^2^Department of Cardiovascular Medicine, Social Insurance Kinan Hospital, 46-70, Shinjyo-cho, Tanabe 646-8588, Japan

## Abstract

*Background*. Recent intravascular ultrasound (IVUS) studies have demonstrated that hypoechoic plaque with deep ultrasound attenuation despite absence of bright calcium is common in acute coronary syndrome. Such “attenuated plaque” may be an IVUS characteristic of unstable lesion. *Methods*. We used optical coherence tomography (OCT) in 104 patients with unstable angina to compare lesion characteristics between IVUS-detected attenuated plaque and nonattenuated plaque. *Results*. IVUS-detected attenuated plaque was observed in 41 (39%) patients. OCT-detected lipidic plaque (88% versus 49%, *P* < 0.001), thin-cap fibroatheroma (48% versus 16%, *P* < 0.001), plaque rupture (44% versus 11%, *P* < 0.001), and intracoronary thrombus (54% versus 17%, *P* < 0.001) were more often seen in IVUS-detected attenuated plaques compared with nonattenuated plaques. *Conclusions*. IVUS-detected attenuated plaque has many characteristics of unstable coronary lesion. The presence of attended plaque might be an important marker of lesion instability.

## 1. Introduction

Intravascular ultrasound (IVUS) is widely used to evaluate vulnerable plaques in patients with coronary artery disease. Attenuated plaque, which is defined as a hypoechoic plaque with deep ultrasound attenuation despite absence of bright calcium, is more often observed in patients with acute coronary syndrome than in those with stable angina [1]. Recent IVUS studies have shown that attenuated plaque is associated with a higher C-reactive protein level, more severe and complex lesion morphology, reduced coronary blood flow before percutaneous coronary intervention, and especially no-reflow after the procedure [2]. However, histopathologic characteristics of attenuated plaque have not been fully investigated. Optical coherence tomography (OCT) is an optical analogue of IVUS that provides high-resolution (10–20 *μ*m) cross-sectional images of coronary arteries. The micron-scale resolution of OCT allows excellent differentiation of atherosclerotic plaque components such as fibrous, fibrocalcific, and lipid [3, 4]. Moreover, OCT can identify vulnerable plaque features including plaque rupture, thrombus, and thin-cap fibroatheroma (TCFA) [5, 6]. Therefore, we used OCT to compare lesion characteristics between IVUS-detected attenuated plaque and nonattenuated plaque.

## 2. Materials and Methods

### 2.1. Study Population

A total of 104 patients with primary unstable angina pectoris (UAP) who had de novo coronary lesions and underwent OCT and IVUS to evaluate culprit lesion morphologies in Wakayama Medical University, Wakayama, Japan, and Social Insurance Kinan Hospital, Wakayama, Japan, were studied retrospectively. UAP was defined according to the Braunwald clinical classification [7, 8]: class I, new onset of severe angina or accelerated angina, no pain at rest; class II, angina at rest within previous month but not within preceding 48 hours; class III, angina at rest within 48 hours. Patients with secondary UAP and postinfarction angina were not included. Patient hospital records were reviewed to obtain information on clinical demographic data and medical history. Hypertension was defined as systolic blood pressure ≥140 mmHg, diastolic blood pressure ≥90 mmHg, or use of antihypertensive drugs. Hypercholesterolemia was defined as a present or past history of low-density lipoprotein-cholesterol level ≥140 mg/dl, or use of statin. Diabetes mellitus was defined as a fasting blood sugar ≥126 mg/dl and haemoglobin A1c ≥6.5%, or use of antidiabetic medications (insulin or oral hypoglycaemics). This study was approved by the institutional review boards of the institutions in which the procedures were performed, and all patients gave written informed consent before cardiac catheterization.

### 2.2. OCT Imaging and Analysis

OCT (the M2 OCT imaging system, LightLab Imaging, Inc, Westford, Mass, USA) was performed before any intervention and IVUS imaging. We used a continuous-flushing (nonocclusive) method for OCT image acquisition. A 0.016-inch wire-type imaging catheter was positioned distal to the culprit lesion. To remove blood cells from the field of view, a mixture of commercially available dextran 40 and lactated Ringer solution was infused from the guiding catheter at 2.5 to 4.5 mL/s with an injector pump. The culprit lesion was imaged with a motorized pull-back device travelling at 1 mm/s. Continuous OCT images were stored digitally for subsequent analysis. The OCT analysis was performed by 2 independent observers who were blinded to the IVUS findings. When there was any discordance between the observers, a consensus reading was obtained. As reported previously [3, 4], fibrous plaques were defined as homogeneous signal-rich regions, fibrocalcific plaques as signal poor regions with sharp borders, and lipidic plaques as signal-poor regions with diffuse borders. If a plaque had both calcification and lipid, the dominant component was adopted for plaque categorization. Plaque rupture was identified by a presence of fibrous-cap discontinuity and a cavity formation in the plaque. Intracoronary thrombus was defined as a protrusion inside the lumen of the artery with signal attenuation [9]. Fibrous cap thickness and lipid size was measured in the lipidic plaques. The fibrous cap thickness was defined as the minimum distance from the coronary artery lumen to the inner border of the lipid core [6]. Lipid size was semiquantified by measuring the lipid arc. OCT-detected TCFA was defined as a plaque with the fibrous cap of <65 *μ*m thick [5]. 

### 2.3. IVUS Imaging and Analysis

**Figure 1:**
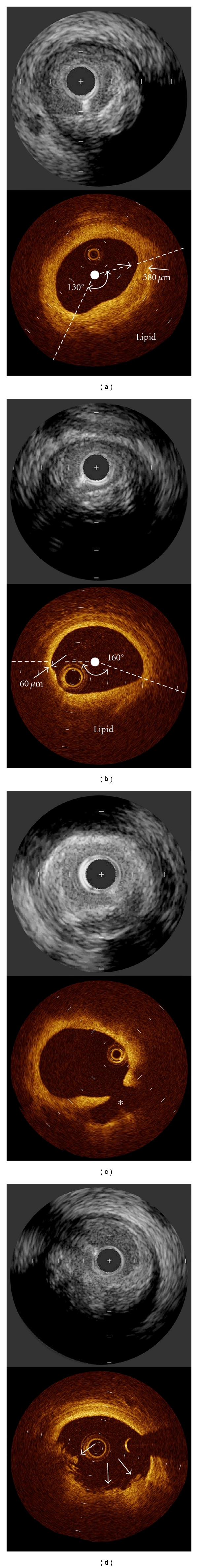
IVUS images of attenuated plaques and corresponding OCT images. OCT revealed that IVUS-detected attenuated plaques include (a) lipidic plaque with fibrous cap of 380 *μ*m thick and lipid arc of 130 degree, (b) thin-cap fibroatheroma with fibrous cap of 60 *μ*m thick and lipid arc of 160 degree, (c) plaque rupture (*), and (d) mural thrombi (arrows).

After intracoronary administration of nitroglycerin (0.2 mg), the IVUS examination was performed using a commercial scanner (Boston Scientific Corporation, Maple Grove, Minn, USA) that consisted of a 40-MHz transducer. The IVUS catheter was advanced beyond the culprit lesion followed by automatic transducer pull back (at 0.5 mm/s) to the aorto-ostial junction. IVUS images were recorded onto a DVD for offline analysis. Using custom-built software, IVUS images were analyzed by 2 independent observers who were blinded to the OCT findings. When there was any discordance between the observers, a consensus reading was obtained. IVUS-detected attenuated plaque was defined as a hypoechoic plaque with deep ultrasound attenuation despite absence of bright calcium [1, 2]. Representative images are shown in Figure 1. To obtain corresponding images of IVUS and OCT, the distances from at least 2 landmarks, such as side branches and/or calcifications, were referred. Quantitative IVUS measurements included external elastic membrane (EEM), lumen, and plaque and media (P&M: defined as EEM minus lumen) cross-sectional area, and plaque burden (defined as P&M divided by EEM) [10]. If the EEM circumference could not be identified because of attenuation, we interpolated the EEM area [2, 11]. The lesion site was defined as the slice with the minimum lumen area. The proximal and distal reference sites were defined as the slices with the most normal looking segments (largest lumen with least plaque) >5 mm proximal and distal to the lesion, but before a major side branch. The remodeling index was calculated as the lesion EEM divided by the mean reference EEM. Positive remodeling was defined as a remodeling index >1.05 [10].

### 2.4. Angiographic Analysis

Quantitative coronary angiography analysis was performed using the Cardiovascular Measurement System (CMS, Medis Medical Imaging System, Leiden, The Netherlands). Angiographic lesion morphology was classified according to the ACC/AHA classification [12]. The percentage of diameter stenosis was measured in the view that was the most severe and not foreshortened.

### 2.5. Statistical Analysis

Statistical analysis was performed using Statview 5.0.1 (SAS Institute, Cary, NC, USA). Categorical variables were presented as absolute numbers and percentages, with comparison using chi square statistics or Fisher exact test, if there was an expected cell value <5. Continuous variables were presented as mean ± standard deviation and were compared using Student's *t*-test. All analyses required a *P* < 0.05 for statistical significance.

## 3. Results

IVUS-detected attenuated plaque was observed in 41 (39%) patients with UAP. Clinical and angiographic characteristics are listed in Table 1. There were no significant differences in coronary risk factors between the patients with attenuated and nonattenuated plaque. Although the frequency of Braunwald clinical class I (20% versus 49%, *P* = 0.002) and class II (7% versus 29%, *P* = 0.011) was significantly lower in the attenuated plaque group compared with the nonattenuated plaque group, the frequency of class III (73% versus 22%, *P* < 0.001) was significantly higher in the attenuated plaque group. Angiographic lesion characteristics were similar between the 2 groups.

IVUS findings are summarized in Table 2. Minimum lumen area (2.5 ± 1.0 mm^2^ versus 2.6 ± 1.0 mm^2^, *P* = 0.676) was similar between attenuated plaques and nonattenuated plaques. At the minimum lumen area site, EEM area (12.4 ± 5.0 mm^2^ versus 10.1 ± 4.4 mm^2^, *P* = 0.016), P&M area (9.9 ± 4.6 mm^2^ versus 7.5 ± 4.0 mm^2^, *P* = 0.006), plaque burden (78 ± 10% versus 73 ± 9%, *P* = 0.022), and frequency of positive remodeling (54% versus 30%, *P* = 0.017) were significantly greater in attenuated plaques compared with nonattenuated plaques.

**Figure 2:**
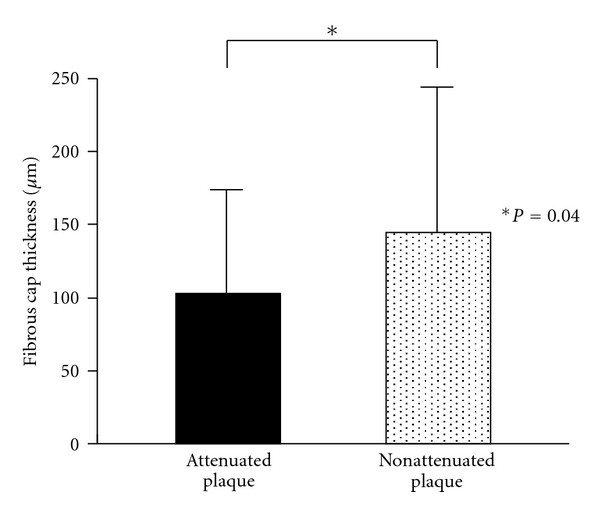
Comparison of fibrous cap thickness between attenuated plaques and nonattenuated plaques. In lipidic plaques, fibrous cap thickness as determined by OCT was significantly thinner in IVUS-detected attenuated plaques compared with nonattenuated plaques.

**Figure 3:**
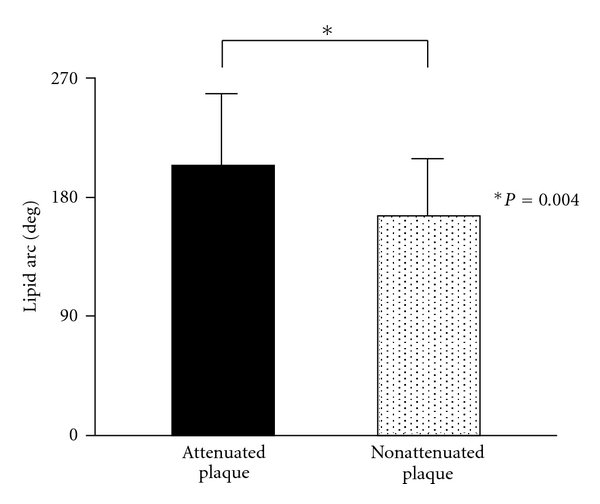
Comparison of lipid arc between attenuated plaques and nonattenuated plaques. In lipidic plaques, lipid arc as determined by OCT was significantly greater in IVUS-detected attenuated plaques compared with nonattenuated plaques.

OCT findings are summarized in Table 3. The frequency of lipidic plaque (88% versus 49%, *P* < 0.001) was significantly higher in attenuated plaques compared with nonattenuated plaques, while the frequency of fibrocalcific plaque (12% versus 42%, *P* = 0.002) and fibrotic plaque (0% versus 9%, *P* = 0.042) was significantly lower in attenuated plaques. In the lipidic plaques, fibrous cap thickness (103 ± 70 *μ*m versus 145 ± 97 *μ*m, *P* = 0.040; Figure 2) was significantly thinner and lipid arc (204 ± 57 degree versus 166 ± 48 degree, *P* = 0.004; Figure 3) was significantly greater in attenuated plaques compared with nonattenuated plaques. OCT-detected TCFA (48% versus 16%, *P* < 0.001), plaque rupture (44% versus 11%, *P* < 0.001), and intracoronary thrombus (54% versus 17%, *P* < 0.001) were more often seen in attenuated plaques compared with nonattenuated plaques.

## 4. Discussion

In this IVUS and OCT study, we demonstrated that IVUS-detected attenuated plaques are associated with lipidic plaque, TCFA, plaque rupture, and intracoronary thrombus. Our results suggest the biologic instability of these lesions.

IVUS is a useful tool for identification of high-risk or unstable lesion morphologies. Previous IVUS data have indicated that culprit lesions in acute coronary syndromes often have a large plaque burden [13, 14], positive remodeling [15, 16], hypoechoic (soft) plaque [17, 18], lipid pool-like plaque characteristics [19, 20], and attenuated plaque [1, 2]. Lee et al. demonstrated that attenuated plaques were more common in patients with ST-segment elevation myocardial infarction than in those with non-ST-segment elevation myocardial infarction (40% versus 18%, *P* < 0.001) and were associated with a higher C-reactive protein level and more complex lesion morphology on angiography [2]. In their study, the location of attenuated plaques was similar to the location of acute occlusions or plaque ruptures, especially with regard to proximal distribution in the left anterior descending coronary artery and right coronary artery [2]. Okura et al. showed that attenuated plaques are related with no reflow and creatine kinase-MB elevation after percutaneous coronary intervention because of distal embolization [1]. Although there is an IVUS study showing attenuated plaques in stable patients [11], most studies support the hypothesis that attenuated plaque is a part of the unstable lesion morphometric spectrum.

OCT has an excellent ability for coronary plaque characterization and vulnerable plaque detection. Histological validation of OCT revealed good intra- and interobserver reliability (*κ* = 0.83–0.84) as well as excellent sensitivity and specificity: 71–79% and 97–98% for fibrous plaques; 95–96% and 97% for fibrocalcific plaques; 90–94% and 90–92% for lipid-rich plaques, respectively [3]. A multimodality imaging study in acute myocardial infarction disclosed superiority of OCT for detection of plaque rupture (73% versus 40% versus 43%, *P* = 0.021) and intracoronary thrombus (100% versus 33% versus 100%, *P* < 0.001) [5]. Moreover, OCT might be the best tool available to detect TCFA in vivo because a good correlation was seen for the measurements of the fibrous cap thickness between OCT and histological examination (*r* = 0.90; *P* < 0.001) [6]. Therefore, we used OCT to evaluate lesion characteristics and demonstrated that IVUS-detected attenuated plaques were associated with lipidic plaque, TCFA, plaque rupture, and intracoronary thrombus. Our results could contribute to the understanding of attenuated plaque and its potential relationship to plaque vulnerability.

Recent studies have shown the mechanisms of ultrasound attenuation in atherosclerotic plaques. In human cadaver coronary arteries, Yamada et al. demonstrated that the percentage of necrotic core area was significantly greater in attenuated plaques than nonattenuated plaques [21]. In line with this histologic study, Wu et al. confirmed in vivo that attenuated plaques were associated with a large amount of necrotic core as determined by virtual histology IVUS imaging [22]. Ito et al. examined directly coronary atherectomy specimens from attenuated plaques and found mature atherosclerosis consisting predominantly of hyalinization, scattered, small areas of calcification, and cholesterol clefts [23]. The random distribution of microcalcification and cholesterol crystals within lipid-rich necrotic core is thought to be responsible for ultrasound wave reflection and dispersion and, as a result, attenuation within the IVUS image [24]. In addition, an animal model has suggested that thrombi with abundant cellular elements could cause backward attenuation due to ultrasound dispersion [25]. The present study showed higher frequency of lipidic plaque and intracoronary thrombus in attenuated plaques compared with nonattenuated plaques. Our result supports the hypothesis that lipid-rich necrotic core of the mature atherosclerotic plaque and intracoronary thrombus maybe important mechanisms of ultrasound attenuation.

## 5. Limitations

There were several limitations. First, this was a retrospective study in nonconsecutive UAP patient. Therefore, the prevalence, clinical feature, and prognostic implication of IVUS-detected attenuated plaque need to be examined by a larger population study. Second, we only included UAP patients. In the examination of attenuated plaque and its potential relationship to plaque vulnerability, our study is limited by the lack of a comparison group with stable presentation. Third, in the IVUS analysis, acoustic shadowing in attenuated plaques may interfere with calculation of remodeling and plaque burden. Fourth, in the OCT analysis, signal attenuation of lipidic tissue or thrombus may preclude visualization and measurement of the entire atherosclerotic plaque. Finally, we used 40-MHz IVUS transducers according to the most previous studies of attenuated plaques. Our results may be not applicable for the IVUS images acquired by other frequency transducers, because the IVUS frequency can affect the degrees of signal penetration (e.g., 20-MHz transducers utilized in virtual histology IVUS have greater penetration than 40-MHz transducers).

## 6. Conclusions

IVUS-detected attenuated plaque has many characteristics of unstable coronary lesion. The presence of attended plaque might be an important marker of lesion instability.

## Figures and Tables

**Table 1:** Clinical and angiographic characteristics.

	Attenuated plaque	Nonattenuated plaque	*P* value
	(*n* = 41)	(*n* = 63)	
Age, y	75 ± 7	73 ± 7	0.169
Male	28 (68)	42 (67)	0.863
Hypertension	33 (80)	44 (70)	0.226
Diabetes mellitus	11 (27)	19 (30)	0.714
Hypercholesterolemia	24 (59)	36 (57)	0.888
Current smoker	15 (37)	15 (24)	0.160

Braunwald clinical classification of UAP			
Class I	8 (20)	31 (49)	0.002
Class II	3 (7)	18 (29)	0.011
Class III	30 (73)	14 (22)	<0.001

Culprit vessel			
LAD	14 (34)	19 (30)	0.669
LCX	7 (17)	8 (13)	0.535
RCA	20 (49)	36 (57)	0.403

ACC/AHA lesion classification			
Type A	8 (20)	18 (29)	0.297
Type B1	17 (41)	24 (38)	0.731
Type B2	11 (27)	13 (21)	0.464
Type C	5 (12)	8 (13)	0.940
Diameter stenosis, %	85 ± 7	83 ± 7	0.178

Values are given as *n* (%) or mean ± standard deviation. UAP: unstable angina pectoris; LAD: left anterior descending coronary artery; LCX: left circumflex coronary artery; RCA: right coronary artery.

**Table 2:** Intravascular ultrasound findings.

	Attenuated plaque	Nonattenuated plaque	*P* value
	(*n* = 41)	(*n* = 63)	
Minimum lumen area site			
EEM area, mm^2^	12.4 ± 5.0	10.1 ± 4.4	0.016
Lumen area, mm^2^	2.5 ± 1.0	2.6 ± 1.0	0.676
P&M CSA, mm^2^	9.9 ± 4.6	7.5 ± 4.0	0.006
Plaque burden, %	78 ± 10	73 ± 9	0.022
Positive remodeling, %	22 (54)	19 (30)	0.017

Proximal reference site			
EEM area, mm^2^	12.5 ± 5.3	10.9 ± 4.4	0.112
Lumen area, mm^2^	8.6 ± 3.7	7.4 ± 3.3	0.117
P&M area, mm^2^	3.9 ± 1.9	3.5 ± 1.3	0.170
Plaque burden, %	31 ± 6	32 ± 6	0.500

Distal reference site			
EEM area, mm^2^	11.4 ± 5.4	9.9 ± 4.4	0.123
Lumen area, mm^2^	8.1 ± 3.8	7.0 ± 3.3	0.133
P&M CSA, mm^2^	3.4 ± 1.7	2.9 ± 1.3	0.144
Plaque burden, %	29 ± 5	29 ± 5	0.974

Values are given as mean ± standard deviation. EEM: external elastic membrane; CSA: cross-sectional area; P&M: plaque and media.

**Table 3:** Optical coherence tomography findings.

	Attenuated plaque	Nonattenuated plaque	*P* value
	(*n* = 41)	(*n* = 63)	
Lesion type			
Lipidic	36 (88)	31 (49)	<0.001
Fibrocalcific	5 (12)	26 (42)	0.002
Fibrotic	0 (0)	6 (9)	0.042
TCFA	20 (48)	10 (16)	<0.001
Plaque rupture	18 (44)	7 (11)	<0.001
Thrombus	22 (54)	11 (17)	<0.001

Values are given as *n *(%). TCFA: thin-cap fibroatheroma.

## Abbreviations

EEM: External elastic membrane
IVUS: Intravascular ultrasound
OCT: Optical coherence tomography
P&M: Plaque and media
TCFA: Thin-capped fibroatheroma
UAP: Unstable angina pectoris.
